# Anaesthetic Management of Secretory Paraganglioma With Cyanotic Heart Disease: Double Trouble

**DOI:** 10.7759/cureus.25328

**Published:** 2022-05-25

**Authors:** Ruma Thakuria, Manpreet Kaur, Rushil Vladimir, Rajeshwari Subramaniam

**Affiliations:** 1 Department of Anaesthesiology, Pain Medicine and Critical Care, All India Institute of Medical Sciences, New Delhi, New Delhi, IND

**Keywords:** autonomic nervous system, cyanotic heart disease, secretory, pulmonary stenosis, atrial septal defect, paraganglioma

## Abstract

Paragangliomas (PGL) in paediatric patients are rarely diagnosed with neuroendocrine tumours. Anaesthetic management of paraganglioma is challenging due to exaggerated haemodynamic alterations. Further associated cardiovascular diseases like congenital cyanotic heart disease (CCHD) with paragangliomas make its management daring and if not properly managed can increase morbidity.

We herein discuss the successful anaesthetic management of a 10-year-old child with paraganglioma and associated atrial septal defect (ASD) with pulmonary stenosis (PS) for adrenalectomy.

Overlapping the clinical spectrum between CCHD and catecholamine-secreting tumour makes the case very challenging. Management of the patient with CCHD and PGL needs a multidisciplinary approach, and intensive vigilance and monitoring are needed for the successful management of such challenging cases.

## Introduction

Paragangliomas are relatively rare tumours originating from chromaffin cells of the autonomic nervous system [1,2]. The incidence of paraganglioma is less than 0.3 cases per million per year and only 10-20% are diagnosed during childhood [3]. Twenty percent of paragangliomas are malignant with a noradrenergic phenotype. The recommended management is complete surgical resection of the tumour [4]. The associated correlation between congenital heart disease and neuroendocrine tumours is statistically significant [5]. Severe hypertension and its sequel make anaesthetic management challenging. We report the perioperative management of surgical resection of paraganglioma in a 10-year-old child with congenital cyanotic heart disease (CCHD).

## Case presentation

A 10-year-old (26 kg, 136 cm) male child presented with high blood pressure (BP) records and was posted for adrenalectomy. He was diagnosed with ASD with severe valvular and subvalvular PS with R-L shunt 3 months earlier and underwent pulmonary valve balloon dilatation resulting in decreased transvalvular gradient (20 mmHg at valvular; 40 mmHg at subvalvular level). For malignant hypertension (BP recordings around 200/130 mmHg), initially, he was treated with injection sodium nitroprusside followed by oral antihypertensive medications (metoprolol 25 mg bid, losartan 25 mg OD, and amlodipine 5 mg BD). He also had a complaint of headache which was persistent in nature for 2-3 hours. Written informed consent for publication was obtained from the parents of the child.

**Figure 1:**
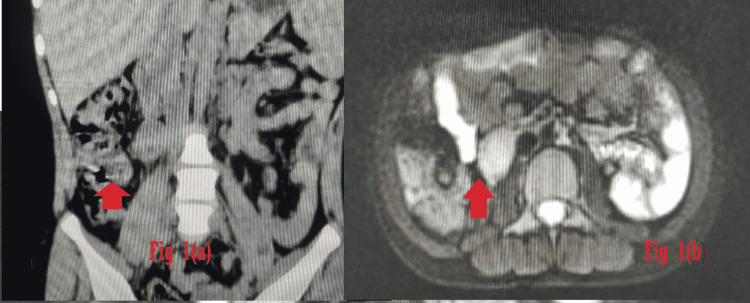
(a) MRI abdomen coronal cross-section view, (b) transverse cut section at L3 level showing hyperintense mass (2 × 1 × 3 cm) in the paracaval location in the inferior pole of the right kidney and anterior to the right psoas muscle.

**Figure 2:**
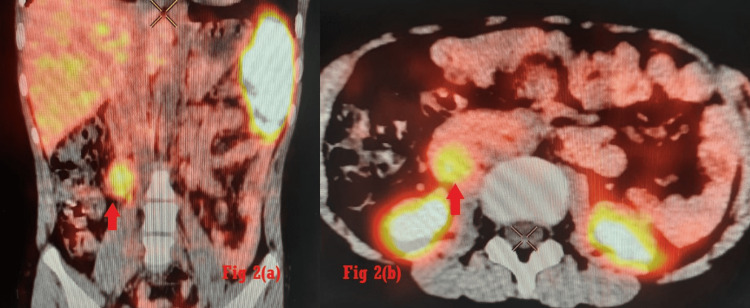
(a) Ga DOTANOC scan coronal view, (b) transverse view at L2-L3 vertebral level. Somatostatin receptor (SSTR) expressing mass (2.1 × 2 × 3 cm) in the right paracaval region anterior to the right psoas muscle, inferior to right kidney.

Investigations revealed raised serum norepinephrine (NE) 720 pg/ml with normal urine catecholamines. Magnetic resonance imaging showed paraganglioma in the right pericaval location adjacent to the inferior pole of the right kidney (Figure 1a-b). PET-CT confirmed the diagnosis (Figure 2a-2b).

He was treated with oral antihypertensives (prazosin 3 mg qid, amlodipine 5 mg bd, and metoprolol 50 mg bd) and tab thyroxine (25 mcg) (thyroid-stimulating hormone (TSH) - 8.94) for two weeks prior to the proposed surgery. On pre-anaesthetic evaluation, he had clubbing and cyanosis, room air saturation was 92%, and blood pressure recordings between 140/110 mmHg and 130/100 mmHg.

There was no complaint of palpitation, sweating, chest pain, pedal enema, or orthostatic hypotension. An electrocardiograph (ECG) showed right ventricular hypertrophy (RVH) and right axis deviation (RAD). Echocardiography showed ASD with R-L shunt and RVH with normal RV function. Biochemical parameters were within normal limits. On the morning of surgery, all antihypertensives were advised to continue. A 20G intravenous (IV) cannula was inserted the evening prior to surgery with EMLA cream.

The child was transferred to the operating room (OR) after premedication with 1 mg midazolam IV. Pre-induction standard ASA (American Society of Anesthesiologists) monitors (ECG, non-invasive blood pressure (NIBP), oxygen saturation (sPO2)) were attached and the vitals were recorded. We used 5-lead ECG with V5 and II with ST analysis due to the elevated risk of intra-operative arrhythmias. The child was administered dexmedetomidine infusion at the rate of 0.5 µg/kg/hr. After preoxygenation, injection magnesium sulphate (MgSO4) 400 mg was administered very slowly over 15 minutes. Anaesthesia was induced with fentanyl 80 µg, etomidate 8 mg, and vecuronium 3 mg. The trachea was intubated with a 6 mm endotracheal tube using a conventional laryngoscope McIntosh size 2 blade with Cormack- Lehane Grade 1 as laryngeal view. He was ventilated with volume control mode (Tidal volume- 8 ml/kg, respiratory rate- 16/min) and anaesthesia was maintained with O2-Air and isoflurane to maintain a minimum alveolar concentration of 0.8-1.0. The right internal jugular vein (IJV) was cannulated and a right radial artery catheter was inserted for the beat-to-beat display of arterial pressure. An epidural catheter was inserted at T11/12 space with loss of resistance at 2 cm and the catheter tip was threaded up to T6 vertebral level (10 cm). At the beginning of surgery, the patient was haemodynamically stable. The rise in BP during tumour handling was controlled with a titrated dose of nitroglycerine (0.1%) administered through pediadrip set as 4-10 mcg boluses. Nitroglycerine boluses were needed till the tumour vessels were ligated. After the tumour vein ligation, noradrenaline infusion was started at the rate of 5-10 µg/kg/min to maintain the BP and was needed till the first postoperative day. The SPO2 level fluctuated between 88-90%. Intraoperative analgesia was provided by 1 µg/kg fentanyl boluses, 500 mg paracetamol, and epidural analgesia with a bolus of 5 ml of 0.125% ropivacaine with 1.2 mg morphine. The surgery lasted for 1 hour and the child was shifted to the high dependency unit (HDU) after ensuring adequate reversal and pain relief. The epidural catheter was maintained for 72 hrs postoperatively with an intermittent bolus of epidural 1.2 mg morphine 12 hourly. The child was closely monitored for hemodynamic alterations, haematocrit and blood sugar, and electrolytes postoperatively. Serum cortisol and urinary catecholamines were within normal limits postoperatively. The postoperative course of the child was uneventful and he was discharged on the fifth postoperative day directly from the paediatric high dependency unit.

## Discussion

Secretory PGL in paediatric patients needs meticulous perioperative management. Perioperative goals in such patients include preoperative adequate alpha- blockade, careful intraoperative monitoring of haemodynamic fluctuations and judicious postoperative monitoring and support.

The index patient was a 10-year-old child with CCHD and secretory pericaval PGL for open resection of the tumour. Literature shows co-occurrence of PGL with CCHD, but the existence of a potential association between these diseases remains controversial [6].

A recent analysis by Opotowsky AR et.al [7] has concluded that there is a strong linkage between CCHD and the development of PGL due to aberrant cellular chronic hypoxia. A higher prevalence of PGL has been found in high altitudes compared to sea levels [8]. Anaesthetic management of such patients is challenging as the patient is exposed to multiple stress points during intubation, tumour manipulation, extubating, and postoperative catecholamine withdrawal. To add to it, the associated CCHD further requires meticulous monitoring.

Preoperative anxiolysis should be one of the goals before induction of anaesthesia. Adequate premedication through either oral or IV route should be administered to the child and the atmosphere of the OR should be calm and quiet. All IV lines should be deaired. We opted for postinduction arterial as he was a child and fear of cannulas could increase the catecholamine surge. However, NIBP was measured every 1 min for the time being. Factors causing the reversal of shunt must be avoided in the perioperative period which includes avoidance of hypercarbia, hypoxia, hypothermia, acidosis, and pain.

Propofol and etomidate are commonly used agents for induction of anaesthesia in PGL resection. But decreased systemic vascular resistance with propofol can result in an increase in shunt flow and a decrease in arterial saturation [9]. Anaesthesia was maintained with isoflurane as isoflurane at minimum concentration has less haemodynamic fluctuations and minimal effect on shunt flow [10].

Ventilation has a vital role in the prevention of an increase in a reversal of shunt flow. High airway pressure ventilation results in a decrease in pulmonary blood flow, an increase in pulmonary vascular resistance (PVR), and an increase in shunt flow causing a significant decrease in arterial saturation.

Good perioperative analgesia decreases perioperative stress and provides pain-free awakening. In the index patient, we threaded the epidural catheter at the thoracic level as it was an open surgical resection of the PGL. After achieving loss of resistance at 2.5 cm, we measured the distance from T11/T12 to T6 using a scale (10 cm) as T6 was the intended site of the incision. Precise placement of an epidural catheter to the thoracic level for continuous perioperative analgesia was the goal. General anaesthesia with epidural analgesia is a safe method in secretory PGL as it prevents fluctuations in hormonal levels as well [11]. For intraoperative surges in blood pressure, short-acting and potent vasodilators like nitroglycerine were preferred over sodium nitroprusside which can cause afterload reduction increasing the R-L shunt.

There is an overlapping clinical spectrum between CCHD and catecholamine-secreting tumours which include hypertension, episodes of palpitation and sweating, headache, dyspnoea on exertion, chest pain, and syncope. The index child was diagnosed with CCHD and detection of PGL was an incidental finding. Catecholamine crises can lead to cardiovascular complications like heart failure, and arrhythmia which further deteriorates in CCHD causing sudden cardiac death [7].

## Conclusions

Management of patients with CCHD and PGL needs a multidisciplinary approach and intensive vigilance, and monitoring is needed for the successful management of such challenging cases.
